# “Too Much Sex and Alcohol”: Beliefs, Attitudes, and Behaviors of Male Adolescents and Young Men Who have Sex with Men in Ghana

**DOI:** 10.2174/1874613601812010069

**Published:** 2018-08-31

**Authors:** Lora L. Sabin, Jennifer Beard, Thomas Agyarko-Poku, Mary DeSilva, Paul Ashigbie, Tami Segal, Michael Esang, Mabel Kissiwah Asafo, Peter Wondergem, Kimberly Green, Samuel Wambugu, Yaw Adu-Sarkodie

**Affiliations:** 1 Department of Global Health, Boston University School of Public Health, 801 Massachusetts Avenue, Crosstown, 3^rd^ Floor, Boston, MA, 02118, USA; 2 Ghana Health Service, Private Mail Bag, Ministries, Accra, Ghana; 3 University of New England, 716 Stevens Avenue, Portland, ME, 04103, USA; 4 Athenahealth, 311 Arsenal Street, Watertown, MA, 02118, USA; 5 Department of Psychiatry & Behavioral Sciences, Nassau University Medical Center, 2201 Hempstead Turnpike, East Meadow, NY, USA; 6 School of Medical Sciences, Kwame Nkrumah University of Science and Technology, Private Mail Bag, University Post Office, KNUST, Kumasi, Ghana; 7 Independent Health Consultant, D 102 Michael Addy Street, Accra, Ghana; 8 PATH, R11.01, 11^th^ Floor, Hanoi Towers, 49 Hai Ba Trung, Hoan Kiem District, Hanoi, Vietnam; 9 MEASURE Evaluation, 123 West Franklin Street, Suite 330, Chapel Hill, NC, 27516, USA

**Keywords:** HIV/AIDS, HIV prevention, Ghana, men who have sex with men (MSM), substance use, transactional sex, qualitative research

## Abstract

**Background:**

Research suggests that men who have sex with men (MSM) often engage in high-risk sex and use illicit substances.

**Objective:**

To increase understanding of HIV knowledge and vulnerability among adolescent and young adult MSM, with a focus on alcohol and drug use and transactional sex.

**Methods:**

We conducted in-depth interviews and Focus Group Discussions (FGDs) with adolescent (aged 15-17 years) and young adult (aged 18-29 years) MSM in Kumasi, Ghana. MSM who reported recent alcohol and/or substance use or engagement in transactional sex were eligible. Questions covered HIV-related knowledge, experiences with substance-use and transactional sex, and attitudes regarding sexual risk-taking and HIV-related services. Data were analyzed thematically using NVivo 10.0 software.

**Results:**

Ninety-nine MSM participated in 44 interviews and 8 FGDs. Most were attending or had completed secondary school. HIV knowledge was high, but with major gaps. Most consumed alcohol; one-fourth used drugs. Alcohol and substances were consumed to enhance pleasure during sex with another man. Transactional sex was common and positively viewed. Half of the participants used condoms inconsistently or never, and self-perceived HIV risk was high. Nearly half faced stigma-related barriers to accessing HIV-related services.

**Conclusion:**

As Ghana strives to achieve the UNAIDS’s 90-90-90 global targets (90% of people living with HIV know their status, 90% who know their status are on sustained treatment, and 90% of those on treatment are virally suppressed), we recommend enhancing MSM-targeted prevention programs, improving care options, and increasing use of critical clinical HIV-services by ensuring that MSM receive unbiased, confidential care.

## 1 INTRODUCTION

More than three decades have passed since HIV was first characterized among men who have sex with men (MSM) in North America [1]. While HIV prevalence has declined globally, MSM continue to be disproportionately affected, particularly in low resource settings [2]. Currently, there are signs of intensifying HIV epidemics among MSM—both in generalized epidemics and where MSM are considered to be at high risk of infection [3]. In sub-Saharan Africa, where the HIV burden is greatest, overall adult prevalence is 4.4%, but typically much higher among MSM [4]. Nowhere is this difference more striking than in West Africa. Adult prevalence in this region is comparatively low—ranging from 1-3% [5]. Yet a number of studies point to far higher infection rates among MSM in Nigeria (35%) [6], Côte d’Ivoire (18%-50%) [7, 8], and The Gambia (10%) [9]. Although a variety of steps have been taken in different countries to expand HIV prevention and treatment programs for MSM [10], this group continues to experience poor access to prevention, testing, and early treatment [10]. Same-sex relationships are illegal throughout the region and discrimination and harassment are widespread, contributing to low uptake of services that are not widely known to be “MSM-friendly” [11].

Adult HIV prevalence in Ghana is estimated at 2% [12] and 17.5% among MSM aged 18-35 years [13, 14]. HIV prevalence among MSM is highest in Accra, the capital (34%), and in Kumasi (13.5%), the second-largest city after Accra [13]. Same-sex relationships are not explicitly illegal in Ghana, but the Criminal Code prohibition of “unnatural carnal knowledge” in Section 104 is widely interpreted as a homosexuality ban [15, 16]. As a result, MSM face widespread discrimination, making them a difficult yet critical population to reach with HIV-related services [10, 17, 18]. Early small studies highlighted low condom use, reluctance to seek care for fear of disrespectful treatment by providers, low rates of testing, and poor access to education on signs of infection [19, 20]. More recently, MSM in Accra, Kumasi, and Manya Korbo reported widespread fear of being outed within the clinic and the community by gossiping clinicians, discriminatory treatment, and ignorance about their specialized health care needs. These concerns were confirmed in the same study through focus group discussions with health care providers [21].

Recent research also suggests high levels of transactional sex and alcohol use, continued low rates of testing, inconsistent condom use, and poor HIV knowledge [22, 23]. Among MSM in Kumasi, the Ghana Men’s Study found that, in the past 12 months, 65% reported drinking alcohol before sex; 20% smoked marijuana; 60% engaged in transactional sex with a man (and 24% with a woman); 26% had five or more male sex partners, and 50% had sex with a woman [13]. At the national level, MSM are estimated to comprise 0.48% (30,979) of the adult male population; of these, approximately 10% (2,994) live in Kumasi [24].

The SHARPER (Strengthening HIV/AIDS Response Partnership and Evidence-based Response) Project was launched with funding from the United States Agency for International Development to address HIV-service access gaps and other vulnerabilities faced by MSM and other key populations [25]. Implemented by FHI360 from 2010-2014, SHARPER worked with community-based organizations to train and deploy peer educators to conduct HIV-prevention outreach. Peer educators were selected for their leadership, and communication skills, and interest and supporting both HIV-negative and -positive peers with HIV education. By 2012, peer educators had reached 12,804 MSM in three regions with most between 15-24 years of age. SHARPER extended outreach efforts in 2013 to contact MSM who had not previously interacted with peer educators [25]. After adding social media approaches, SHARPER connected with nearly 30,000 MSM from October 2012 through September 2013, approximately 92% of the MSM population [26, 27].

Ghana is committed to meeting the HIV prevention and treatment needs of MSM and sex workers. This focus on understanding and meeting the needs of key populations is central to Ghana’s goal to achieve the United Nation’s 90-90-90 targets by 2020: 90% of people living with HIV (PLHIV) know their status, 90% on sustained antiretroviral treatment, and 90% viral suppression [12]. This study was one of nine USAID-funded qualitative research projects designed to better understand the needs of key populations and improve HIV prevention and support interventions. Other studies in the project assessed needs of young female sex workers, prisoners, people who inject drugs, women who work in bars, post-secondary female students, people living with HIV, and older (aged 30 years and above) MSM [28-34].

The research reported here was designed to contribute policy- and program-relevant information about MSM in urban Ghana. In particular, we focused on two important and possibly overlapping behaviors of young MSM: use of alcohol and illicit drugs, and transactional sex. The specific study objectives were to explore the following among adolescent and young adult MSM: 1) the extent of substance use and transactional sex, 2) beliefs and attitudes related to substance use and transactional sex, and 3) HIV knowledge and related risk behaviors. To our knowledge, this is the first qualitative study to focus on young MSM in Ghana who engage in two related high-risk activities: transactional sex and/or alcohol and drug use.

## 2 MATERIAL AND METHODS

### 2.1 Study Population and Inclusion Criteria

We conducted In-Depth Interviews (IDIs) and Focus Group Discussions (FGDs) with adolescent (aged 15-17 years) and young adult (aged 18-29 years) males who reported male-to-male sex in the previous 12 months. To be eligible for the study, participants had to meet one of the following criteria: 1) alcohol or illicit drug use, defined as self-reported use of an average of two or more alcoholic drinks per day for at least two days per week, in the most recent month, or any quantity of an illicit substance such as marijuana, cocaine, methadone, amphetamines, and glue in the most recent month; and 2) engagement in transactional sex, defined as self-reported sex with another male in exchange for money, gifts, or favors in the previous six months.

### 2.2 Sample Size and Sampling

We interviewed adolescent and young adult MSM who fit at least one of the following groups: a) young adult MSM who met criteria for substance use; b) adolescent MSM who met criteria for substance use; c) young adult MSM who met criteria for transactional sex; and d) adolescent MSM who met criteria for transactional sex. To supplement these interviews, we facilitated eight FGDs (two discussions for each of the above groups).

Study participants were identified and recruited using a snowball sampling approach. Initially, we recruited eight MSM aged 15-29 years in Kumasi who received services at a public medical clinic, and another eight MSM in the same age range who participated in SHARPER programs. Each “index participant” was asked to introduce the study to one to three peers. Participants engaged in either an interview or FGD, but not both.

### 2.3 Data Collection

Data were collected in June and July 2012 by research assistants trained by Boston University, Kwame Nkrumah University of Science and Technology, and FHI360. All research assistants had previous experience working with marginalized populations, including MSM. Each interview and FGD was conducted by a team of two research assistants in which an interviewer, who was from the MSM community, posed initial and follow-up questions while another took notes. Interviews and FGDs were conducted in either English or Twi (based on participant preference) in a safe, convenient location. Each activity used a previously pilot-tested, semi-structured question guide, took sixty to ninety minutes to complete, and was audio-recorded.

Interview questions focused on individual knowledge of HIV, beliefs, attitudes, and behaviors. Queries covered types of substances used, the frequency and context of use, attitudes about substance use, and links between substance use and risk-taking. Further questions addressed the extent and context of transactional sex, how such sex was perceived, and specific behaviors such as condom and lubricant use. Interviewers probed for information about first sexual experiences, HIV knowledge, perception of HIV risk, health concerns, and access to HIV services in Kumasi.

The FGDs covered the same topics described above, but questions were posed about participants’ peers or MSM in general. While the in-depth interviews addressed the amount of alcohol an individual consumed, the FGDs asked about what was typical among friends and acquaintances. Background information, including age, education, and marital status, was collected from all participants.

The study was approved by the institutional review boards of Boston University Medical Center and the Kwame Nkrumah University of Science and Technology. Adult participants provided verbal informed consent to participate; adolescents aged 15-17 years provided verbally informed assent. To ensure privacy, no identifying information was collected.

### 2.4 Data Analysis

Audio-taped discussions were transcribed into written English, and transcripts were transferred to Boston in encrypted, password-protected files. Transcripts were coded and analyzed using NVivo 10.0 software [35]. Themes were developed inductively in response to patterns that emerged in the data. We compared responses across age categories (older *vs*. younger MSM) and study activity (in-depth interview *vs*. focus group discussion). Although FGD questions focused on peer experiences, many participants shared personal experiences and perspectives. These experiences were all included in the analysis. Responses were prioritized by the number who shared similar views or behaviors, although divergent statements were also explored. We identified direct statements for illustrative purposes.

## 3 RESULTS

### 3.1 Characteristics of Participants and Description of First Sexual Experience

Ninety-nine MSM age 15 to 29 participated in the study. Participant characteristics and socio-demographic data are reported in Table **1**.

Most participants reported that their first sexual experience was with another male, occurring between the ages of 9-23 years. The vast majority (97 of 99) reported that their first partners were older men; one-half were school connections, either an older student, a classmate, or a teacher. One in four participants said that sex had been forced on them, in each case by an older male. Alcohol, marijuana, money and gifts were often the impetus for voluntary sex. Most reported receiving money or gifts.

Our analysis revealed numerous themes related to HIV knowledge, views of and engagement in high-risk sex, and challenges related to accessing medical services, including HIV-related services. These results are provided below. Direct statements are provided where appropriate, with longer statements presented in Table **2**. Differences are noted between participants of different age groups (adolescents *vs*. young adults) and study activity (in-depth interviews *vs*. focus group discussions).

### 3.2 Knowledge of HIV

The majority of all participants knew the basics of HIV and its means of transmission, although the young adults tended to have a higher degree of knowledge. Among adolescents, roughly half demonstrated substantial knowledge, identifying multiple routes of transmission and sometimes describing the biological basis of HIV infection and how it attacks the immune system. Other means of infection reported were: “deep kissing,” “accidents,” and “from a salon with infected combs and instruments.” Nearly one-half learned about HIV/AIDS at school, while more than one-third learned about it through mass media, particularly television. See Table **2** for illustrative statements.

When asked about HIV prevention, nearly all participants mentioned condom use, while some brought up avoiding “sharing of sharps” with an infected person, abstinence, being faithful to one’s partner(s), and reducing the number of partners. HIV treatment was poorly understood. Although many participants understood the need to go to a healthcare facility for care and treatment, only a minority knew about the existence of Anti-Retroviral Therapy (ART). Participants in four of the eight FGDs and nearly one-fourth of those interviewed appeared to be unaware of HIV treatments.

### 3.3 Alcohol and Drug Use

Most participants said that they and their peers drank alcohol, with no differences between age groups. Although amounts varied, the most typical was 2-4 bottles (usually containing 625 milliliters) of beer per day. One young adult described drinking 20 bottles of beer in a single day. Most claimed that their main motivation for drinking was to make sex more enjoyable. As a 27 year-old FGD FGD participant explained: “When you drink before having sex, it makes you enjoy the sex because it increases your sexual drive or feelings.” Additional reasons were to reduce inhibitions and embarrassment, improve mood, and enable a wider range of sexual positions. Close to one-half believed that alcohol use was unrelated to condom use during sex. Others were divided. Some believed that alcohol use reduced the probability of condom use by reducing inhibitions and impairing one’s ability to assess risks. As one FGD participant, aged 24 years, explained: “One of my partners refuses to use a condom whenever he is drunk. I prefer he smokes to drinking alcohol.” Others—particularly young adults—voiced the opinion that, if one was truly committed to condom use, availability of a condom would determine condom use, not the effect of alcohol.

A minority (one-fourth) of participants revealed that they and their peers used drugs or illicit substances. Marijuana was by far most popular, followed by “poppers” (alkyl nitrites) and cocaine. No participant discussed injection drug use. Most men who used drugs reported that sex was more enjoyable because of increased energy, sexual drive, and stamina. This typical statement was made by an 18-year-old: “Yes, [sex is] more enjoyable. …It makes me happy during sex”. Most felt that drug use was unrelated to condom use; however, some stated that drugs reduced it.

### 3.4 Transactional Sex

Nearly all participants indicated that transactional sex was common among MSM in Kumasi. Most interview participants had engaged in transactional sex in the prior year, estimating that it occurred “often” or “many times a week.” Typical descriptions were: “I have sex for money at all times” and “[It is] very common especially (among) students.” Although both young adult and adolescent MSM reported commonly receiving something in return for sex, young adults were more likely than adolescents to also give money, gifts or favors to sex partners.

Most study participants agreed that engaging in transactional sex, whether on the giving or receiving end, was acceptable. Many felt that money was the main motivation, and for some, it was an additional or sole source of income. Several asserted that money was the primary or even the only reason some men had sex with other men: “I do that [have sex with men] for money and favors and am sure that is the reason why most of us are involved,” explained an FGD participant, aged 16 years. One 23-year-old FGD participant said that many MSM expected to pay always: “Some people think that if they do not give money, you will not agree [to have sex].” Some participants revealed a sense of shame at engaging in transactional sex and recognized the risks of unprotected sex. As one 17-year-old IDI participant acknowledged: “I feel bad because it is a situation I wish I could have avoided. …. It is bad to have sex in exchange for money. If the person is infected with HIV you might end up with the disease as well.” In spite of this, many stressed the high financial reward and believed it was common among their peers to be persuaded by money to have unprotected sex. Almost one-third of the men interviewed and most in five of the eight FGDs believed transactional sex increased the likelihood of sex without a condom. Almost all IDI participants who engaged in transactional sex said their partners did not use condoms consistently, with them or with other lovers.

### 3.5 Condom Use

Most participants claimed to use condoms, although slightly over one-half reported doing so consistently. Consistent condom use was more typical among young adults than adolescents. Inconsistent condom-users gave two main rationales. In IDIs, the most common reason for inconsistent condom use was that protection was unnecessary with trusted partners. As one 17-year-old IDI participant explained: “I usually use it on those I don’t know.” Among FGD participants, the most commonly cited reason for an inconsistent condom was reduced sexual enjoyment: “We actually don’t use condoms because they give less pleasure.” Additional reasons for non-use of condoms were: unavailability when needed, intoxication (and forgetting), agreeing to have unprotected sex with a handsome man or a paying client, and coercion. Other reasons for not using condoms included the need to negotiate use with partners, difficulty obtaining them, difficulties putting them on, poor fit, and discomfort.

### 3.6 Lubricants

Most participants reported that lubricant use during sex was widespread. All but six said they used some form of lubricant, usually water-based, regularly. The reasons given for using lubricants focused on making sex more enjoyable and less painful. Most commented that lubricants make penetration easier (83%). Others noted that lubricants help prevent cuts and condom breaks, ease condom use and withdrawal, and quicken ejaculation. Explanations for non-use included: lack of need (condoms were already lubricated, or saliva could be used instead), lack of knowledge on where to get them, and feeling too ashamed to ask about lubricants.

### 3.7 Perception of HIV Risk

Most participants believed they were at risk of HIV, noting unprotected sex as the primary risk factor for themselves and their peers. Some participants felt at risk of HIV infection for reasons other than unprotected sex. These included: kissing, sharing blades, visiting barber shops, and (more generally) having sex with other men. As one young adult noted: “It is risky …. If one protects [oneself] by using condoms, you can still contract [HIV] through kissing.”

### 3.8 Perceived Health Problems and Access to Services

Few participants reported specific health problems. Typical responses to health-related questions included: “I don’t have any health problems” and “No, I am fit.” Among those noting concerns, the most common complaints were weakness or headaches after sex and sexually-transmitted infections. Other concerns were pain during urination, genital rashes, genital discharge, anal warts, anal pain, anal bleeding after sex, gonorrhea, and exposure to a partner’s blood.

The most common challenge to staying healthy was unprotected sex. Directly or in response to related questions, half of all participants reported concerns about unprotected sex. In one adolescent FGD, when queried about the greatest difficulties their peers faced in staying healthy, participants responded: “Too much sex and alcohol”; “Too much sex”; “Not practicing personal hygiene”; “Too much unprotected sex, too much drugs and alcohol”; “Too many outings and too much alcoholism”; “Having unprotected sex.”

Nearly one-half of participants described challenges accessing HIV testing, treatment, or referral services due to discrimination or ill-treatment by providers. Participants mentioned both fear and personal experience of discrimination. Stigma was noted as a barrier to seeking general health services and HIV testing services specifically. A typical comment, by a 29-year-old FGD participant, was: “It is not easy at all. The way and manner a certain nurse talked to us, it was embarrassing, so we felt uncomfortable. Because of that, we left the hospital.” Additional challenges were poor service quality, mistrust of test results, lack of funds/health insurance, inadequate time with providers, and shame. Half of the participants highlighted that “friendly staff” at some facilities facilitated access. Several mentioned new policies at some facilities which either let certain physicians attend specifically to MSM or designated special days of the week for providing MSM-friendly services.

When asked about needed services, participants most frequently mentioned treatment for sexually transmitted infections and HIV counseling, testing, and treatment. Another recommendation was creating a comprehensive, MSM-friendly clinic. One adolescent FGD participant noted: “A special center for gays will reduce a lot of the problems that we go through.” Others stressed the need for shorter wait times.

## 4 DISCUSSION

This study was designed to illuminate beliefs and behaviors contributing to vulnerability to HIV infection faced by young men who have sex with men in Ghana, particularly those engaging in transactional sex and alcohol or drug use. Several important findings contribute to our understanding of this population and suggest ways to reduce their risk of HIV infection. First, general HIV knowledge was high. Most participants were able to describe the basics of transmission and prevention, and the importance of condom and lubricant use. This is more positive than findings from the Ghana Men’s Study, which found knowledge levels of around 50% [14]. Our qualitative approach, which was iterative and flexible in the data gathering process, may have permitted the collection of more nuanced information than is typically collected *via* quantitative surveys.

Most study participants, particularly adolescents, knew little about HIV treatment and pre-exposure prophylaxis. This finding is consistent with previous studies and highlights that, while substantial progress has been made in recent years in educating MSM about HIV [25, 26], more education is needed targeting youth and emphasizing treatment options and pre-exposure prophylaxis.

Second, although drug use was relatively limited, alcohol consumption was widespread. High levels of daily alcohol use were common throughout the sample. While alcohol use among adult MSM has already been documented [14, 23], ours is the first study to highlight substantial alcohol consumption among adolescent MSM in Ghana, and to provide evidence on a primary rationale for such high use: that alcohol is consumed to increase pleasure during sex. Many participants acknowledged the risks associated with mixing alcohol with sex. Perhaps because of their age and more extensive experience, older participants in our sample were particularly vocal about the need to commit to condom use. Their emphasis on the heightened risk when condoms were not available underscores the importance of ongoing and expanded efforts to ensure that MSM have consistent access to condoms.

Third, we found that frequent transactional sex was common among young MSM. Most participants reported engaging in transactional sex and feeling comfortable doing so, viewing the exchange of money, gifts, and favors as a natural and positive activity. This is not surprising in a low-resource environment, given evidence from Ghana and elsewhere that young people are often persuaded or inclined to exchange sex for money or goods [13, 28, 32, 36, 37]. The Ghana Men’s Study found participation in transactional sex among MSM aged 18-35 years to be 60% in Kumasi and 65% nationwide [13].

We also found substantial overlap between substance (alcohol and drug) use and transactional sex activity, with those who engaged in transactional sex describing higher alcohol consumption than those who did not. This suggests a possibly higher risk of inconsistent condom use among young MSM who engage in transactional sex. Participants frequently expressed concern that transactional sex increased the risk of unprotected sex, mainly due to pressure or enticement. Most who engaged in transactional sex acknowledged that their sex partners included men who had other sex partners and that condom use in these partners’ other encounters was unknown. This indicates that, in addition to making condoms more accessible, the expansion of training programs focused on improving negotiating condom use is important to improve the effectiveness of MSM-oriented HIV programs.

Fourth, nearly one-half of the participants reported using condoms inconsistently or never. This confirms other reports of inconsistent condom use among MSM in Ghana. The Ghana Men’s Study found condom use rates of 64% (Kumasi) and 65% (nationwide) at last sex [13]. One of the main rationales given, in addition to reduced sexual pleasure, was that a condom was not necessary during sex with a trusted partner. Most of our study participants also reported regular lubricant use (86% of interviews), exceeding levels of consistent use (69%) found in Tanzania [38]. This is promising, and an important contribution to the limited data available on lubricant use among MSM in Africa.

Fifth, we found that accessing needed health services is a substantial challenge for many MSM in Ghana due to the fear of discrimination by providers. This is not surprising, as previous research has revealed low rates of HIV testing and poor access to other services among MSM in Ghana [19, 20, 23]. In a recent qualitative study in Accra, Kumasi, and Manya Korbo, MSM reported high levels of anxiety about seeking health care services for the following reasons: fear that providers would tell others in the clinic or in the community about their sexual orientation, disrespectful or other discriminatory interactions, lack of knowledge about MSM health needs, refusal to provide needed services (including genital and anal physical examinations), and overlooking clinical symptoms [21]. Focus group discussions with MSM and health care providers also revealed differing perceptions of quality of care. Many clinicians stated the importance of providing non-discriminatory care. But patients reported receiving useful advice delivered without empathy. Even in situations where MSM said they were received in a way that was friendly, they sometimes felt that their concerns were ignored and reported having to endure lectures about their sexual partners and mannerisms.

We found evidence of improved access at some facilities, and this is confirmed by some favorable reviews of health care services in the Accra, Kumasi, Manya Korbo study [21]. Further efforts focused on enhancing the ability of medical staff to be more sensitive to the needs of MSM are needed to improve HIV and other health services. In addition, using “peer-navigators,” MSM who help peers to contact specially-trained and trusted medical staff, has potential to improve the use of HIV-related services [39].

The findings from this study were used by the SHARPER project to tailor outreach and services to teenage and young adult MSM. This was done through recruiting and training adolescent and youth MSM peer educators, and by developing a social media-based HIV prevention strategy [26]. Training for MSM youth included strategies to stop or reduce alcohol and illicit drug use, improve negotiation skills related to condom use and transactional sex, and increase HIV prevention and treatment literacy. These peers also distributed condoms and lubricants to their friends and acquaintances. Further, SHARPER worked with this population to identify and train relevant healthcare facilities and providers to welcome MSM and other key populations and provide them with high-quality, judgment-free care. SHARPER project staff also developed a referral guide for peers listing MSM-friendly health services [26].

This study had several limitations. First, some participants may have provided biased information—possibly from a desire to please facilitators (moderator-acceptance bias); to avoid expressing conflicting views in the company of other FGD participants who held strong opinions (dominant-respondent bias); or because of poor recall. To minimize bias and gather a wide range of perspectives, we used a variety of probes to triangulate data and two forms of data collection (in-depth interviews and focus group discussions). Second, the research was conducted in one city only; our findings may not represent the views and experiences of young Ghanaian MSM beyond Kumasi. Third, the sample size for this study was relatively small at 99 (48 adolescents and 51 young adults). The alignment between our qualitative findings, the qualitative findings of Kushwaha *et al*. in their study of health services, and with the much larger, quantitative Ghana Men’s Study bolsters confidence in our findings.

## CONCLUSION

HIV prevention among MSM has been a high priority in Ghana, as is evidenced by the SHARPER project and other efforts by the government and development partners. Despite these efforts, we found gaps in HIV-related knowledge, multiple high-risk behaviors, and stigma-related barriers to accessing health care among young MSM in Kumasi. As Ghana strives to achieve the 90-90-90 goals set by UNAIDS, we recommend focusing HIV prevention efforts among adolescent and young adult MSM in the following critical areas: alcohol use before sex, access to condoms, condom negotiation, and knowledge of treatment options. Access to HIV-services will improve by training MSM-friendly service providers and expanding the use of “peer navigators”.

## Figures and Tables

**Table 1:** Socio-demographic characteristics of IDI and FGD participants.

	In-Depth Interviews Number (%) *(N=44)*	Focus Group Discussions Number (%) *(N=55)*
**Age group**		
Adolescents	19 (43.2)	29 (52.7)
Young Adults	25 (56.8)	26 (47.3)
**Years of age**		
14-17	19 (43.2)	26 (47.3)
18-20	12 (27.3)	9 (16.4)
21-24	4 (9.1)	11 (20.0)
25-29	9 (20.5)	9 (16.4)
**Marital status**		
Single	38 (86.4)	50 (90.9)
Cohabitating	6 (13.6)	5 (9.1)
**Education^φ^**		
None	0 (0.0)	2 (3.6)
Primary	2 (4.5)	0 (0.0)
Secondary	41 (93.2)	50 (90.9)
Tertiary	1 (2.3)	3 (5.5)

**^φ^** Highest level of education attained.

**Table 2:** Illustrative statements from participants.

***First sexual experience*** *He acted as a woman by showing me a porn video, which aroused me. I initially disapproved of it, but he kept on pushing and eventually enticed me with money. I later agreed and it was because money was involved.* – Adolescent, IDI participant *Well, he [a teacher] … took me to his house and gave me a drink. I didn’t see anything but I woke up and realized he had had sex with me and he warned me not to tell anyone.* – Adolescent, IDI participant *He drugged me. I saw it, but I was not able to control myself.* – Adolescent, IDI participant *He approached me and said he liked me. So I asked him, ‘How?’ And he replied by saying he was interested in me. He promised me a lot of things, especially money. It was also a time that I needed money, so I had to agree when he [propositioned] me.* – Adolescent, IDI participant **Alcohol and drug use** *Sex and alcohol are brothers; they [have sex] indiscriminately when they get drunk.* – Young adult, FGD participant *Alcohol makes you enjoy sex, because you don’t feel any pain during sexual intercourse.* – Young adult, FGD participant *[I drink] to release the same [bad feelings] people heap on us.* – Adolescent, IDI participant *No, I always use a condom to have sex. Drinking alcohol cannot prevent me from using a condom. I always have my senses when I drink alcohol.* – Young adult, FGD participant *Alcohol douses your senses and can impair your condom use.* – Adolescent, FGD participant **Perception of HIV risk** *It is risky... Because if one protects [oneself] by using condoms, you can still contract [HIV] through kissing.* – Young adult, FGD participant *One needs to use protection; otherwise, [sex] can be dangerous for HIV.* – Young adult, IDI participant *It is serious especially when people do it [have sex] without the use of condoms, and it usually happens when we are in need of money.* – Adolescent, FGD participant *Yes. [I believe I am at a high risk of infection.] This is because I sometimes don't protect myself when I have sex with my partner because I trust him, even though I do not know what he can do behind [my back].* – Adolescent, IDI participant *Those who need money may go raw if it’s the only option--and that will put them at risk of contracting HIV.* – Young adult, FGD participant	***Transactional sex*** *Sometimes I ask how I got this far, as I wish I had not been doing it. But the money and trips to expensive hotels can be exciting.* – Adolescent, IDI participant *I do that [have sex] for money and favors, and am sure that is the reason why most of us are involved. So it happens most often.* – Adolescent, FGD participant *I like sex and as a man, I know that I have to pay them [other men].* – Young adult, FGD participant *I feel okay because I am rendering a service.* – Adolescent, FGD participant *Some people think that if they do not give money you will not agree [to have sex with them].* – Adolescent, FGD participant *The problem of unemployment made me enter into gay sex for pay.* – Young adult, FGD participant **Condom use** *Sometimes some guys get drunk such that they do want to use condom.* – Adolescent, FGD participant, *If these drugs enhance sex, then [they] could make you forget to use a condom, if you already have a problem with condom use.* – Young adult, FGD participant *Most people are interested in the money and so they don’t care if they have sex without protection.* – Young adult, IDI participant *I don’t think drugs can influence my decision on condom use.* – Young adult, FGD participant *Yes, I met one client who is older than me and after agreeing and having sex, he refused to pay because he wanted to go raw and I made him use condom… [he] beat me in addition.* – Adolescent, FGD participant **Challenges accessing health services** *Some of the staff in these facilities know clients who are gay and so they stigmatize and criticize us when we go there to seek health care.* – Adolescent, FGD participant *I usually face these challenges: discrimination, mistrust, poor staff attitude and client relation(s), and long waiting time.* – Adolescent, IDI participant *We do not know [where to access] HIV services in Kumasi.* – Young adult, FGD participant *One of the major difficulties is stigmatization.* – Adolescent, FGD participant *I want the place [health facility] to be free of people so I can feel comfortable to go there. I need confidentiality to be ensured in [places] providing health services.* – Young adult, FGD participant
